# Frequency of facial touching in patients with suspected COVID-19 during their time in the waiting room

**DOI:** 10.1017/ice.2020.389

**Published:** 2020-08-03

**Authors:** Eduardo Perez-Alba, Laura Nuzzolo-Shihadeh, Alejandro Fonseca-Ruiz, Gloria Mayela Aguirre-García, Marco Antonio Hernández-Guedea, Edelmiro Perez-Rodriguez, Adrián Camacho-Ortiz

**Affiliations:** 1 Servicio de infectología, Hospital Universitario Dr. José Eleuterio González, Universidad Autónoma de Nuevo León, Monterrey, Mexico; 2 Departamento de Epidemiología Hospitalaria y Servicio de Infectología, Hospital Universitario Dr. José Eleuterio González, Universidad Autónoma de Nuevo León, Monterrey, Mexico; 3 Subdirección de Asistencia Hospitalaria, Hospital Universitario Dr. José Eleuterio González, Universidad Autónoma de Nuevo León, Monterrey, Mexico; 4 Dirección Hospitalaria, Hospital Universitario Dr. José Eleuterio González, Universidad Autónoma de Nuevo León, Monterrey, Mexico

*To the Editor—*Coronavirus disease 2019 (COVID-19) is a novel disease caused by severe acute respiratory coronavirus virus 2 (SARS-CoV-2), which has spread worldwide.^1^ Viral transmission is suspected to occur through droplets produced predominantly while coughing and sneezing.^2^ Alternatively, viral particles may remain infectious in inert surfaces and act as fomites.^3^

Although transmission by droplet aspiration and contact with other respiratory secretions are well described as contagion mechanisms, face touching has not been as extensively discussed.^4–6^ As universal masking gains popularity among healthcare professionals (HCPs), the fact that they promote face touching must not be forgotten.^7^ Despite the latter, face masks have a crucial role in protection, but whether they provide protection for patients in the outpatient setting is unknown.

This phenomenon could be crucial in the transmission of SARS-CoV-; thus, we explored the frequency of face touching in patients with possible COVID-19 awaiting evaluation in an ambulatory clinic.

## Methods

We designed and implemented a study in which video cameras were installed in the waiting room of a respiratory infection diagnosis unit during March 2020. As patients waited for care their behavior was recorded and later logged. Upon arrival to the clinic, all subjects were instructed to use a face mask and to perform hand hygiene using alcohol-based hand rub.

Widely visible signs were used to notify those present about the video surveillance in the waiting room for research purposes; however, they were not notified of the purpose of the study. Patients were monitored from their entrance to the clinic and until they left. The study was performed in the respiratory infections diagnosis unit at University Hospital “Dr. José Eleuterio González” in Monterrey, Mexico. We included all adult patients who received medical attention in the clinic during the study period. We excluded pediatric patients and other vulnerable populations.

Our main objective was to determine the number of times that patients with suspected COVID-19 touched their faces and their face masks during their time in the waiting room. Age, gender, cell phone use, time spent in the waiting room, and test results were also registered. The local ethics committee approved the study (no. IF20-0008).

The study population was characterized using descriptive statistics to determine measures of central tendency. A sample size of 45 patients was calculated with a 95% confidence interval and a 0.5 standard error. We used SPSS version 22.0 software (IBM, Armonk, NY) for the statistical analyses.

## Results

In total, 350 patients were recorded during the study period. We analyzed a random sample of 98 patients that could be clearly visualized and followed during their stays. The average patient age was 37 years (range, 18–77), and the total recorded time was 880 minutes. The average length of stay was 49 minutes (range, 12–97), including time in the waiting area and medical attention. In total, 62 of 98 patients were already wearing a face mask when arrived at the unit, and 25 patients put on a face mask according to the instructions provided at the entrance of the unit. Only 11 patients did not wear a mask during their time in the clinic.

On average, a patient with a face mask touched his or her face 11.41 times (range, 0–80) compared to 11.38 for a patient without a mask (range, 0–29; *P* = .49). A study participant adjusted his or her face mask an average of 7.4 times (range, 0–31) and used a cell phone a mean of 0.2 times during his or her stay (range, 0–1).

Of the 98 subjects, only 5 (5.1%) had a positive RT-PCR test for SARS-CoV-2. These patients touched their faces an average of 9.9 times (range, 1–29), which was not statistically significant (*P* = .74). Results adjusted per hour are provided in Table 1.

**Table 1.** Comparison Between Detected and Nondetected SARS-CoV-2–Positive Patients

Patient Characteristic	SARS-CoV-2 Detected (n = 5)	SARS-CoV-2 Not Detected (n = 93)	*P* Value
With face mask	4	87	.315
Without facemask	1	6	.315
No. of face touches per hour	15.59 (1.7–31.1)	13.32 (0–78.3)	.748
No. of facemask adjustments per hour	5.32 (0–4.4)	10.77 (0–60)	.601
No. of phone calls per hour	0.4 (0–2)	0.21 (0–5)	.623

## Discussion

SARS-CoV-2 is thought to be transmissible in close contact by aerosols and contaminated surfaces.^3^ We describe the complexity of controlling simple strategies such as avoidance of face touching in an outpatient care setting. Although wearing a face mask may be useful in preventing droplet generation, frequent face touching may represent a disadvantage. The outer surface of medical masks may become contaminated while used, and as such, may create a fomite that facilitates contagion at the time of removal.^8^

Considering that only 5 of the analyzed patients had COVID-19, it is probable that other viruses could be causing the patient’s symptoms. One of our patients touched his face 80 times during his stay in the unit, while another adjusted the face mask 31 times. These observations bring into question whether the overall risk of SARS-CoV-2 contagion for our patients was mitigated by this kind of PPE and whether frequent face touching may lead to the dissemination of other respiratory viruses.

PPE that includes a face mask may be useful for HCPs for several reasons. Importantly, it is used alongside other PPE, which may protect infected droplets from entering the eyes.^7^ Also, HCPs are probably better trained at doffing PPE and may have a smaller risk of hand contamination. Thus, we wonder whether the benefits of patients wearing face masks while awaiting medical attention outweigh the risks and reinforce the need for adequate hand hygiene and environmental disinfection, especially in high-risk areas.

Masks may induce a false feeling of safety in patients, making them potentially harmful. We suggest that in the outpatient scenario in times of COVID-19 or other respiratory infections, the use of masks by patients should be accompanied by media or personnel notifications so that they know that avoiding face touching may be as, if not more important than, wearing the mask.
